# Nuclear Receptor ERRγ Protects Against Cardiac Ischemic Injury by Suppressing GBP5‐Mediated Myocardial Inflammation

**DOI:** 10.1096/fj.202500763R

**Published:** 2025-07-10

**Authors:** Junhao Qiu, Qianji Che, Yichao Zhang, Mu Chen, Zhixing Wei, Yangjinming Bai, Tingting Zhao, Ji Yan, Zhengyang Wu, Zhentao Fei, Yuepeng Wang, Qian Wang, Yi‐Gang Li

**Affiliations:** ^1^ Department of Cardiology, Xinhua Hospital, School of Medicine Shanghai Jiao Tong University Shanghai China

**Keywords:** ERRγ, GBP5, inflammation, myocardial infarction, pyroptosis

## Abstract

Myocardial inflammation plays a critical role in the progression of injury following myocardial infarction (MI), yet the transcriptional mechanisms regulating cardiomyocyte inflammation to mitigate post‐ischemic injury remain poorly understood. This study elucidated the role of Estrogen‐Related Receptor Gamma (ERRγ) in modulating the inflammatory response post‐MI, demonstrating that ERRγ expression was downregulated in ischemic tissue and hypoxic neonatal mouse ventricular myocytes (NMVMs). Cardiomyocyte‐specific overexpression of ERRγ reduced infarct size, improved cardiac function, and suppressed excessive myocardial inflammation and pyroptosis by binding to the GBP5 promoter, thereby inhibiting GBP5 transcription and reducing NLRP3 inflammasome assembly. The protective effects of ERRγ overexpression were reversed by overexpressing GBP5, and the ERRγ agonist DY131 also improved cardiac function after MI. These findings suggest that ERRγ activation reduces myocardial ischemic injury by regulating cardiomyocyte inflammation and pyroptosis, highlighting ERRγ as a potential novel therapeutic target for attenuating post‐MI injury.

## Graphical abstract

Cardiomyocyte‐specific overexpression of ERRγ after myocardial infarction improves cardiac function by suppressing the expression of its pro‐inflammatory downstream target GBP5, thereby inhibiting inflammasome assembly, alleviating myocardial inflammation, and pyroptosis.

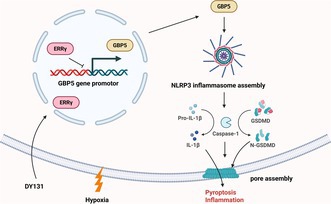

**Abbreviations**
ASC: apoptosis—associated speck—like protein containing a CARD
CASPASE1: apoptosis‐related cysteine peptidase1
DAMP: danger‐associated molecular pattern
DPN: diarylpropionitrile
ERRγ: estrogen related receptor γ
ERs: estrogen receptors
GBP5: guanylate‐binding protein 5
GSDMD: gasdermin domain‐containing protein 1
IL: interleukin
LAD: left anterior descending
LVEDV: left ventricular end‐diastolic volume
LVEF: left ventricular ejection fraction
LVESV: left ventricular end‐systolic volume
LVFS: left ventricular fractional shortening
LVIDd: left ventricular internal dimension in diastole
LVIDs: left ventricular internal dimension in systole
MI: myocardial infarction
NLRP3: nucleotide‐binding oligomerization domain‐containing protein‐like receptor protein 3
NMVMs: neonatal mouse ventricular cardiomyocytes
PPARs: peroxisome proliferator‐activated receptors
PPT: propyl‐pyrazole‐triol
PRR: pattern recognition receptors
RNA‐seq: RNA sequencing
TEM: transmission electron microscopy
TNF‐α: tumor necrosis factor‐α
WT: wild type

## 1 Introduction

Myocardial infarction (MI) continues to exhibit high morbidity and mortality rates worldwide and remains the predominant manifestation of coronary artery disease [1, 2]. Given the limited proliferative capacity of cardiomyocytes, the loss of cardiomyocytes due to no or delayed revascularization might result in irreversible cardiac dysfunction [3]. Therefore, salvaging necrotic cardiomyocytes has become a primary goal post‐MI.

In the early stages of MI, prolonged ischemia results in extensive cardiomyocyte death and the release of numerous danger signals, termed danger‐associated molecular patterns (DAMPs). DAMPs bind to their cognate pattern recognition receptors (PRRs) expressed on surviving parenchymal cells and infiltrating leukocytes, thereby activating the NLRP3 inflammasome [4]. NLRP3 interacts with an adaptor protein, apoptosis‐associated speck‐like protein containing a caspase recruitment domain (ASC), which subsequently recruits Pro‐caspase‐1, promoting its autocatalytic activation [5, 6]. Activated caspase‐1 not only facilitates the maturation of pro‐inflammatory cytokines IL‐1β and IL‐18, amplifying the inflammatory response, but also cleaves GSDMD. The resulting NT‐GSDMD interacts with phosphatidylinositol in the plasma membrane, forming pores that lead to cell swelling, membrane rupture, and the release of inflammatory cytokines—a process termed pyroptosis [7, 8].

Nuclear receptors are known to be important controllers of the NLRP3 inflammasome pathway [9]. ERRγ is a member of the ERR subfamily within the orphan nuclear receptor family, and it plays a key role in coordinating gene expression involved in various cellular processes [10]. ERRγ is expressed in multiple tissues, with particularly high expression in energy‐demanding organs such as the heart. Previous studies have demonstrated that ERRγ is critical for regulating cellular metabolism and mitochondrial energy synthesis [11]. For instance, ERRγ inhibits the growth of esophageal squamous cell carcinoma by suppressing the glycolytic enzyme PKM2 [12]. In renal tubular cells, ERRγ ameliorates acute kidney injury by mediating TFAM expression and improving mitochondrial function [13]. Recent studies have also linked ERRγ to inflammatory responses in various diseases. In a mouse model of LPS‐induced acute liver injury, intraperitoneal injection of DY131 (an ERRγ agonist) attenuated hepatic inflammation, oxidative stress, and apoptosis [14]. Additionally, ERRγ conditional knockout mice exhibit pancreatic inflammation, fibrosis, and acinar cell death, mimicking features of pancreatitis [15]. However, it remains unknown whether ERRγ can mitigate myocardial inflammation, reduce pyroptosis, and improve cardiac function in the context of MI. Further investigation is required to elucidate the role and molecular mechanism of ERRγ in these processes.

Guanylate‐binding protein 5 (GBP5) is a member of the interferon‐γ (IFN‐γ)‐inducible guanosine triphosphatase (GTPase) family [16], known to participate in cellular inflammatory responses. Previous studies have shown that GBP5 interacts with the pyrin domain of NLRP3, promoting the assembly of the NLRP3‐ASC complex and enhancing NLRP3 inflammasome formation [17]. Moreover, overexpression of GBP5 in mice exacerbates LPS‐induced sepsis‐associated liver injury through activation of the NLRP3 inflammasome [18]. Additionally, GBP5 has been shown to promote the expression of pro‐inflammatory cytokines and chemokines in immune cells via NLRP3 inflammasome activation [19]. These findings suggest a crucial role of GBP5 in inflammatory diseases. However, the relationship between GBP5 and MI‐induced inflammatory responses remains to be elucidated.

In our study, we aimed to elucidate whether targeting ERRγ can modulate the inflammatory response and pyroptosis in cardiomyocytes following ischemic injury. Additionally, we sought to demonstrate the underlying mechanism by searching the impact of ERRγ on GBP5 and NLRP3 inflammasome formation.

## 2 Materials and Methods

### 2.1 Animals

Adult male C57BL/6J mice (weight: 21 ± 2 g) (RRID:IMSR_JAX:000664) were purchased from Shanghai Yaokang Biotechnology Co. Ltd. and housed at the Animal Center of Xinhua Hospital, affiliated with Shanghai Jiao Tong University School of Medicine, under a 12‐h light/dark cycle. This study involves a total of 288 mice. A total of 43 mice died either during the MI model establishment process or through the intervention observation period, which will not be included in the final statistical analysis. For animal studies, we totally conducted 4 experiments. Experiment 1: To investigate the temporal expression pattern of ERRγ protein in the infarct border zone and infarct area following MI, mice were randomly divided into Sham, 1 day post‐MI, 3 days post‐MI, and 7 days post‐MI; Experiment 2: To evaluate the effects of ERRγ overexpression on post‐MI cardiac function, inflammation, cardiomyocyte pyroptosis, and infarct size, mice were used to randomly assign to four groups: Sham + AAV9‐cTNT‐NC, Sham + AAV9‐cTNT‐ERRγ, MI + AAV9‐cTNT‐NC, and MI + AAV9‐cTNT‐ERRγ; Experiment 3: To investigate the compensatory effect of GBP5 overexpression on ERRγ‐induced changes in post‐MI cardiac function, inflammation, cardiomyocyte pyroptosis, and infarct size, mice were used to randomly assign to four groups: Sham + AAV9‐cTNT‐GFP, MI + AAV9‐cTNT‐GFP, MI + AAV9‐cTNT‐ERRγ and MI + AAV9‐cTNT‐ERRγ + AAV9‐cTNT‐GBP5; Experiment 4: To investigate the therapeutic effect of the ERRγ agonist DY131 on MI mice, mice were used to assign to four groups randomly: Sham + PBS, Sham + DY131, MI + PBS, and MI + DY131. All animal experiments conformed to the Guide for the Care and Use of Laboratory Animals published by the US National Institutes of Health. The study was approved by the ethics committee of Xinhua Hospital affiliated to Shanghai Jiao Tong University School of Medicine.

### 2.2 Mouse Model of MI

Mice were anesthetized by 2% isoflurane inhalation and mechanically ventilated with a rodent respirator device. After left thoracotomy, the left anterior descending (LAD) coronary artery was ligated with 6–0 silk sutures. A successful occlusion of the vessel was confirmed by immediately turning pale in the LAD coronary artery perfusion area and dynamic changes of electrocardiogram (ECG). 5–0 silk sutures were used to close the chest and skin. Sham‐operated mice were subjected to the similar procedure without ligation.

### 2.3 Echocardiography Analysis

Mice at 7 days post‐MI were anesthetized with 1% isoflurane inhalation and placed on a heated pad to maintain 37°C body temperature. The ejection fraction (EF), fractional shortening (FS), left ventricular end‐systolic dimensions (LVSD) and left ventricular end‐diastolic dimensions (LVDD) were measured from M‐mode images of echocardiography (Vevo 3100, FUJlFILM, Shanghai, China) with a 30 MHz transducer.

### 2.4 Western Blotting and Quantitative Real‐Time qPCR

Proteins from the cardiac ventricular tissue of mice and cultured NMVMs were extracted by RIPA that contained protease inhibitors and phosphatase inhibitors. An equal quantity of protein (20–40 μg) was resolved by SDS/PAGE and transferred to PVDF membranes. The blots were blocked with 5% non‐fat milk in TBS buffer with 0.5% Tween‐20 for at least 2 h at room temperature and then incubated with antibodies against ERRγ (1:1000, HUABIO, ER61910), GBP5 (1:1000, Abcam Cat# ab313390, RRID:AB_3677678), GSDMD (1:500, Abcam Cat# ab219800, RRID:AB_2888940), NLRP3 (1:500, Abcam Cat# ab263899, RRID:AB_2889890), Caspase1 (1:1000; Proteintech Cat# 22915‐1‐AP, RRID:AB_2876874), β‐actin (1:1000, ABclonal Cat# AC038, RRID:AB_2863784). Then, the blots were incubated with primary antibody at 4*°*C overnight (about 12 h). The blots were then incubated with secondary antibodies conjugated with horseradish peroxidase for 1–2 h at room temperature. The Gel Imaging System (Tanon) and Image J software were used to image and analyze the intensity of each band normalized to loading control β‐actin. Total RNA was extracted from cardiac ventricular tissues and cultured cells using TRIzol (Takara, Cat# RR036A). Total RNA (1000 ng) was reversely transcribed into cDNA using the Prime‐ScriptTMRT reagent kit (Takara, RR036A). qRT‐PCR was performed using SYBR green (Q711; Vazyme, Nanjing, China) and normalized to β‐actin expression. ABI QuantStudio 3 (Applied Biosystems) with standard PCR conditions (95*°*C for 30 s, followed by 40 cycles of 95*°*C for 10 s and 60*°*C for 30 s) was used to run the samples.

### 2.5 Tissue Immunofluorescence

On the third day after MI or sham surgery, mice were deeply anesthetized. Hearts were rapidly excised, fixed in 4% paraformaldehyde, embedded in paraffin, and sectioned at a thickness of 4 μm. The sections were placed in an antigen retrieval box containing EDTA buffer (pH 8.0) and heated in a microwave at medium power for 8 min, followed by an additional 10 min at low power. Subsequently, the sections were permeabilized with 0.1% Triton X‐100 for 20 min and blocked with 3% bovine serum albumin (BSA) for 30 min at room temperature. For immunofluorescence staining, mouse heart sections were stained with primary antibodies against anti‐ERRγ antibody (1:200, Novus Cat# NBP2‐45698, RRID:AB_3309828), anti‐cTNT antibody (1:200, Proteintech Cat# 15513‐1‐AP, RRID:AB_2206563), and anti‐F4/80 (1:200, Cell Signaling Technology Cat# 30325, RRID:AB_2798990), which were then incubated overnight at 4°C. After washing off the primary antibodies, appropriate secondary antibodies were added and incubated for 60 min at room temperature in the dark. The sections were then stained with DAPI for 10 min at room temperature in the dark and mounted using an anti‐fade mounting medium. All images were captured with an Olympus microscope (Olympus, Japan) or CaseViewer (3DHISTECH Ltd., Budapest, Hungary) and measured by Image J software (National Institute of Health, Bethesda, Maryland, USA).

### 2.6 Infarct Size Measurement

The method used to process the heart for the measurement of infarct size was similar to that previously described [20]. After euthanizing mice at 3 days post‐MI under deep anesthesia with pentobarbitone sodium, the hearts were immediately excised and frozen at −40°C for 20 min. Once slightly thawed at room temperature, the hearts were sectioned into 2–3 mm slices using a very sharp blade, ensuring neat cutting. The heart sections were then placed in a 12‐well plate (careful not to compress the tissue) and immersed in TTC staining solution. The plate was placed in a 37°C water bath, and the tissue was stained in the dark for approximately 30 min. Every 10 min, the tissue was gently agitated to ensure even staining. Finally, the sections were fixed in 4% paraformaldehyde. Red areas indicated viable tissue, while white areas represented infarcted regions.

### 2.7 Transmission Electron Microscope

At Day 3 post‐MI, several tissue blocks (~1 mm^3^) from the infarct border zone were immediately harvested and fixed in 2.5% glutaraldehyde in 1.5 mL (or 2 mL) centrifuge tubes at 4°C for 12–24 h. The samples were then post‐fixed with 1% osmium tetroxide for 1–2 h. After carefully removing the osmium waste, the samples were rinsed three times with 0.1 M phosphate‐buffered saline (PBS, pH 7.4), each for 15 min. Subsequently, the tissues were dehydrated through a graded ethanol series (30%, 50%, 70%, and 90%) for 15 min at each concentration, followed by two changes of 100% ethanol for 20 min each. The samples were then dehydrated twice in 100% acetone for 20 min each. After dehydration, the tissues were infiltrated and embedded using a mixture of acetone and embedding resin. Pure embedding resin was poured into embedding molds, and the samples were positioned within the resin. The molds were then polymerized in a 70°C oven for 12–48 h. Once hardened, the resin blocks were trimmed and sectioned into ultrathin slices (70–90 nm) using an ultramicrotome. Sections were collected on copper grids and stained with uranyl acetate for 8–15 min, followed by lead citrate staining for 5–10 min. After drying, the sections were examined using a transmission electron microscope (TEM, HITACHI HT7800), and representative images were captured for analysis.

### 2.8 Construction of Recombinant Adenovirus, Adeno‐Associated Virus, and Small RNA Interference

Adenovirus expressing ERRγ were conducted by the OBiO Technology Corp. Ltd. (Shanghai, China) and expressing GBP5 were conducted by the WZ Biosciences Inc. For ERRγ and GBP5 overexpression in mice, the entire coding region of the mouse ERRγ and GBP5 cDNA was subcloned into the pcAAV9‐cTNTo‐3xFLAG‐P2A‐GdGreen‐WPRE vector downstream of the cardiac troponin‐T promoter. Mice were given adeno‐associated virus (3.0 × 10^11^ Vg/mouse) via tail intravenous injection. Cardiac‐specific ERRγ and GBP5 overexpression was successfully induced 4 weeks after AAV9 transfection in adult C57BL/6 J mice. The corresponding control mice received pcAAV9‐cTNTo‐3xFLAG‐P2A‐GdGreen‐WPRE vector injections at the same dose, respectively.

ERRγ, GBP5, and selective siRNAs and negative controls were designed and synthesized by Shanghai Generay Biotech Co. Ltd. Control siRNA was employed as a negative control under similar conditions. siRNAs used in our experiment were: mouse ERRγ siRNA sequence: sense: GAUUGUCUCGCAUUUGUUGGU antisense: CAACAAAUGCGAGACAAUCUU; mouse GBP5 siRNA sequence: sense: AAAGUGAGCAAUAUACCUGGGTT antisense: CCCAGGUAUAUUGCUCACUUUTT. siRNA transfection was conducted using Lipofectamine 3000 reagent (Thermo Fisher Scientific, USA).

### 2.9 Cell Transfection

The ERRγ overexpression adenovirus (Adv‐ERRγ) was conducted by the OBiO Technology Corp. Ltd. (Shanghai, China) and the GBP5 overexpression adenovirus (Adv‐GBP5) was conducted by the WZ Biosciences Inc. For ERRγ overexpression experiment in vitro, the NMVMs were infected with Adv‐ERRγ that was added in 1 mL DMEM (low glucose) containing 10% FBS in a 3.5 cm dish for 24 h; then the medium was replaced with 2 mL fresh DMEM (low glucose) containing 10% FBS and cultured for another 24 h. For the GBP5 rescue experiment in vitro, the NMVMs were infected with Adv‐ERRγ in the same way as above for 24 h; then the medium was replaced with 1 mL DMEM (low glucose) containing Adv‐GBP5 in a 3.5 cm dish for 24 h, and the medium was replaced with 2 mL fresh DMEM (low glucose) containing 10% FBS and cultured for another 24 h.

For ERRγ knocking down experiment in vitro, siRNA against ERRγ was transfected in NMVMs at a final concentration of 50 nM using Lipofectamine 3000 reagent (Thermo Fisher Scientific, USA). Cells were collected for the experiments after 48 h of transfection.

### 2.10 RNA Sequencing

Total RNA was isolated from NMVMs treated with Adv‐ERRγ or Adv‐GFP in the context of hypoxia (*n* = 3 in each group) using the mirVana miRNA Isolation Kit (AM1561; Thermo Fisher Scientifc, Waltham, MA). kaiaoK5500Spectrophotometer (Kaiao, Beijing, China) and RNA Nano 6000 Assay Kit of the Agilent Bioanalyzer 2100 system (Agilent Technologies, CA, USA) were used to evaluate the RNA purity, concentration, and integrity. The libraries were generated using the NEB Next Ultra RNA Library Prep Kit for Illumina (#E7530L, NEB, USA) following the manufacturer's recommendations, and index codes were added to attribute sequences to each sample. Low‐quality reads of the raw data were abandoned, and all the analyses were based on high‐quality clean data. Results with *q* value < 0.05 and |log2 (fold change)| > 3 were assigned as differentially expressed genes (DEGs), and Kyoto Encyclopedia of Genes and Genomes (KEGG) pathway enrichment, Gene Set Enrichment Analysis (GSEA) and Gene Ontology (GO) were performed subsequently.

### 2.11 Isolation and Culture of Neonatal Mouse Ventricular Cardiomyocytes

NMVMs were isolated from 3‐day‐old neonatal C57BL/6J mice. After sacrifice, hearts were excised and flushed with dPBS to get rid of any remaining blood. The ventricles were cut into 1 mm pieces and washed with dPBS twice. Then, the fragments were transferred to a 10 cm dish containing 0.25% trypsin and dPBS overnight at 4°C. The next day, the fragments were re‐suspended in about 14 mL DMEM (low glucose) and supplanted with 1 mL collagenase type II (Worthington, LS004176). The tissue and the suspensions were shaken in 37°C water for approximately 17 min to separate cardiomyocytes and fibroblasts. After that, the cell suspensions were combined, filtered using sterile techniques, and plated on uncoated culture dishes for 1 h. After 1 h, the cell suspensions were collected and plated on culture dishes. After 48 h, the medium was replaced to remove the dead cells. NMVMs were maintained in low glucose DMEM medium supplemented with 10% FBS and 1% penicillin/streptomycin (P/S). The medium was changed every other day.

### 2.12 Dual‐Luciferase Reporter Assay

The promoter region (−2000 to +50) of GBP5 was cloned into pGL3‐Basic luciferase reporter vector (Promega). HEK293T (Abcam Cat# ab255593, RRID:CVCL_0063) cells were co‐transfected with the luciferase reporter vector, *Renilla* luciferase reporter plasmid, and experimental vectors. After 48 h, luciferase activity was detected by the Dual‐Luciferase Reporter Assay System (Promega) according to the manufacturer's instructions. *Renilla* luciferase activity served as a control for normalization.

### 2.13 Data Analysis

Data were analyzed using Graph‐Pad Prism 9.0 or SPSS 19.0 statistical software and represented as mean *±* SD or percentage of at least four independent experiments. Two‐tailed Student's tests were used for two‐group comparisons, and ANOVA followed by post hoc Tukey's test was used for multiple‐group comparisons. A value of *p* < 0.05 was considered statistically significant.

## 3 Result

### 3.1 Downregulation of ERRγ in the Ischemic Myocardium

**FIGURE 1:**
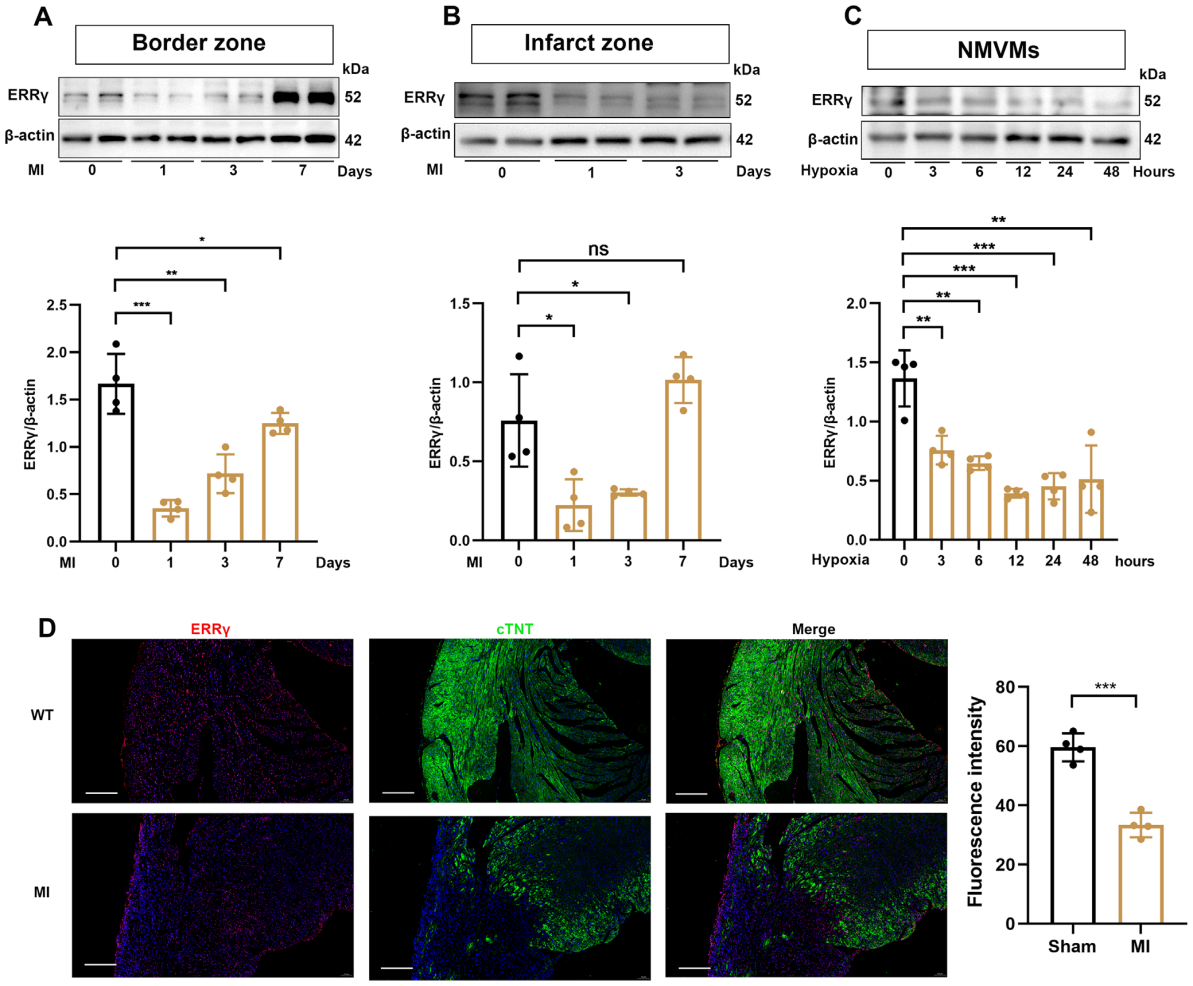
ERRγ expression is downregulated following MI. (A, B) Western blot analysis of ERRγ levels in the border zone and infarct zone at Days 1, 3, and 7 post‐MI in mice, compared to sham controls, with corresponding quantification. *n* = 4. (C) ERRγ protein expression in neonatal mouse ventricular myocytes (NMVMs) at 0, 3, 6, 12, 24, and 48 h post‐hypoxia. *n* = 4. (D) Immunofluorescence co‐staining of ERRγ, cTnT, and DAPI in MI hearts. *n* = 4. Scale bar = 100 μm. Data are presented as mean ± SD. Data in (A–C) were analyzed by one‐way ANOVA with Bonferroni's multiple comparison test. Data in (D) were analyzed by two‐tailed Student's *t*‐test. *p < 0.05, **p < 0.01, ***p < 0.001.

To elucidate the functional roles of ERRγ in MI, we established a mouse MI model and analyzed the temporal protein expression patterns of ERRγ in WT MI mice. Immunoblotting revealed that ERRγ expression decreased in the border zone up to Day 7 post‐MI (Figure 1A). A similar trend was observed in the infarct zone (Figure 1B). In vitro experiments further demonstrated that ERRγ protein expression decreased with prolonged hypoxia, reaching its lowest level at 12 h in neonatal mouse ventricular myocytes (NMVMs) exposed to simulated ischemia by glucose and oxygen deprivation up to 48 h (Figure 1C). Moreover, immunofluorescent analysis showed that ERRγ expression was significantly lower in the infarct border zone of MI mice compared to sham‐operated mice at Day 3 post operation (Figure 1D). These findings indicate that ERRγ is downregulated post myocardial injury.

### 3.2 Cardiac‐Specific Overexpression of ERRγ Improved Left Ventricular Function and Decreased Ischemic Injury

**FIGURE 2:**
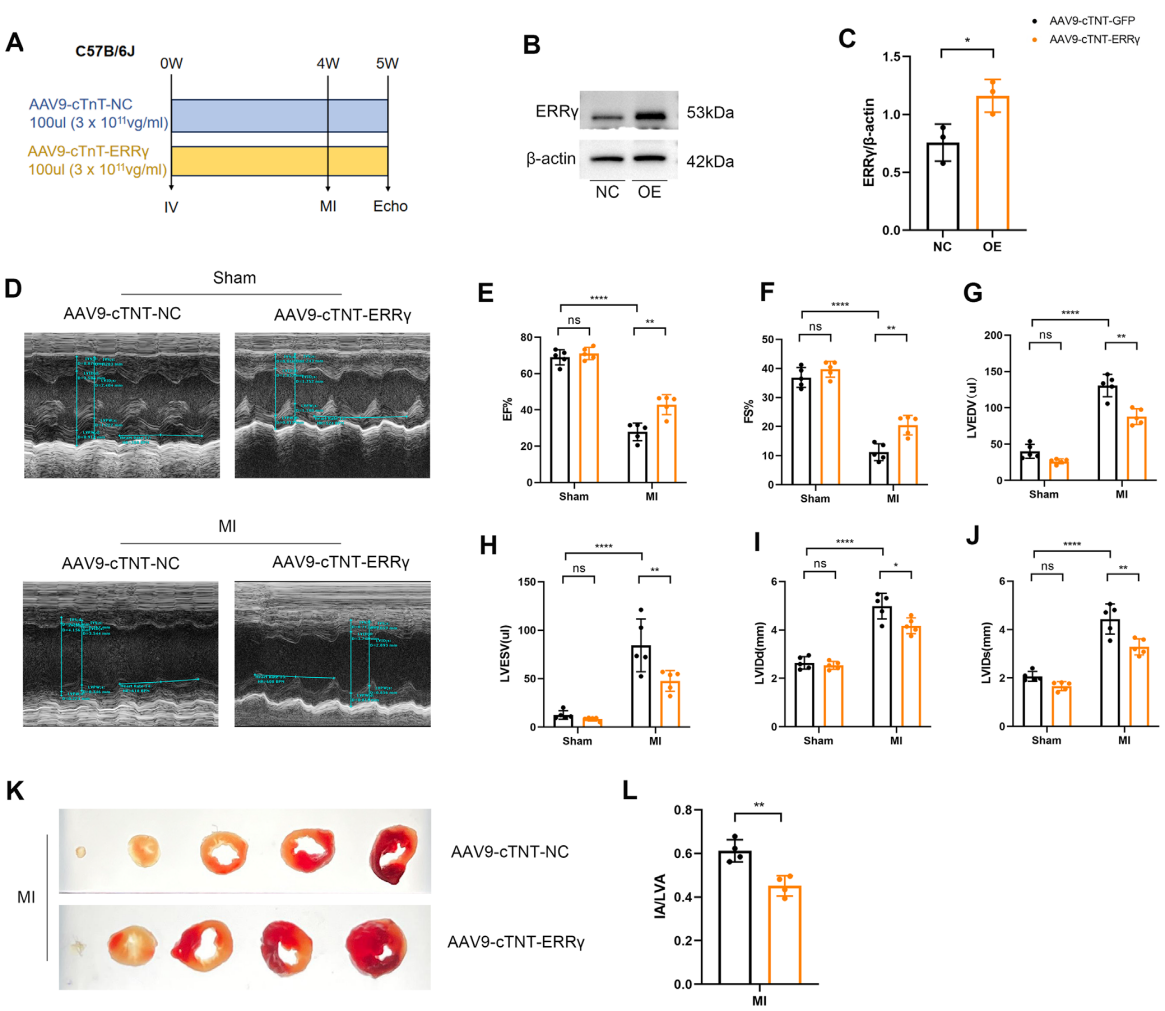
ERRγ attenuates cardiac injury following MI. (A) Experimental timeline and design for AAV9‐cTnT‐GFP and AAV9‐cTnT‐ERRγ administration in C57BL/6J mice. (B, C) ERRγ expression levels were assessed in AAV9‐cTnT‐GFP and AAV9‐cTnT‐ERRγ infected mice using WB, with corresponding quantification. *n* = 3. (D) Representative echocardiography images of MI mice 7 days post‐infection with AAV9‐cTnT‐GFP or AAV9‐cTnT‐ERRγ. (E–J) Echocardiographic analysis of LV ejection fraction (LVEF), fractional shortening (FS), left ventricular end‐diastolic volume (LVEDV), left ventricular end‐systolic volume (LVESV), left ventricular end‐diastolic dimension (LVIDd), and end‐systolic dimension (LVIDs). *n* = 4. (K, L) Infarct size quantified by TTC staining as a percentage of ventricular area (post MI 3 day). *n* = 4. Data are presented as mean ± SD. Data in (E–J) were analyzed by one‐way ANOVA with Bonferroni's multiple comparison test. Data in (C, L) were analyzed by two‐tailed Student's *t*‐test. *p < 0.05, **p < 0.01, and ****p < 0.0001.

Next, we examined the impact of ERRγ overexpression (OE) on myocardial injury induced by MI. A cardiac‐specific ERRγ overexpression mouse model was constructed via tail vein injection of AAV9 virus (Figure 2A). Four weeks later, the efficiency of overexpression was validated, and western blot results indicated that ERRγ expression levels were significantly higher in the OE group compared to the NC group, confirming effective viral overexpression (Figure 2B,C, Figure S1C). Subsequently, permanent LAD ligation was performed, and echocardiographic assessment was performed at Day 7 post‐MI. Echocardiographic results showed that left ventricular ejection fraction (LVEF) and left ventricular fractional shortening (LVFS) were significantly improved in the ERRγ OE MI mice compared to the control MI mice (Figure 2D–F). Additionally, left ventricular end‐diastolic volume (LVEDV) and end‐systolic volume (LVESV) (Figure 2G,H), as well as left ventricular end‐diastole diameter (LVIDd) and end‐systole diameter (LVIDs) (Figure 2I,J) were significantly reduced. Assessment of infarct size using Triphenyltetrazolium chloride (TTC) staining demonstrated a significant reduction in infarct area in ERRγ OE MI mice compared to control virus MI mice (Figure 2K,L).

In summary, cardiac‐specific overexpression of ERRγ improves cardiac function and ameliorates ischemic damage in MI mice.

### 3.3 ERRγ Overexpression Alleviates Inflammation and Pyroptosis in Cardiomyocytes Under Hypoxic Conditions

**FIGURE 3:**
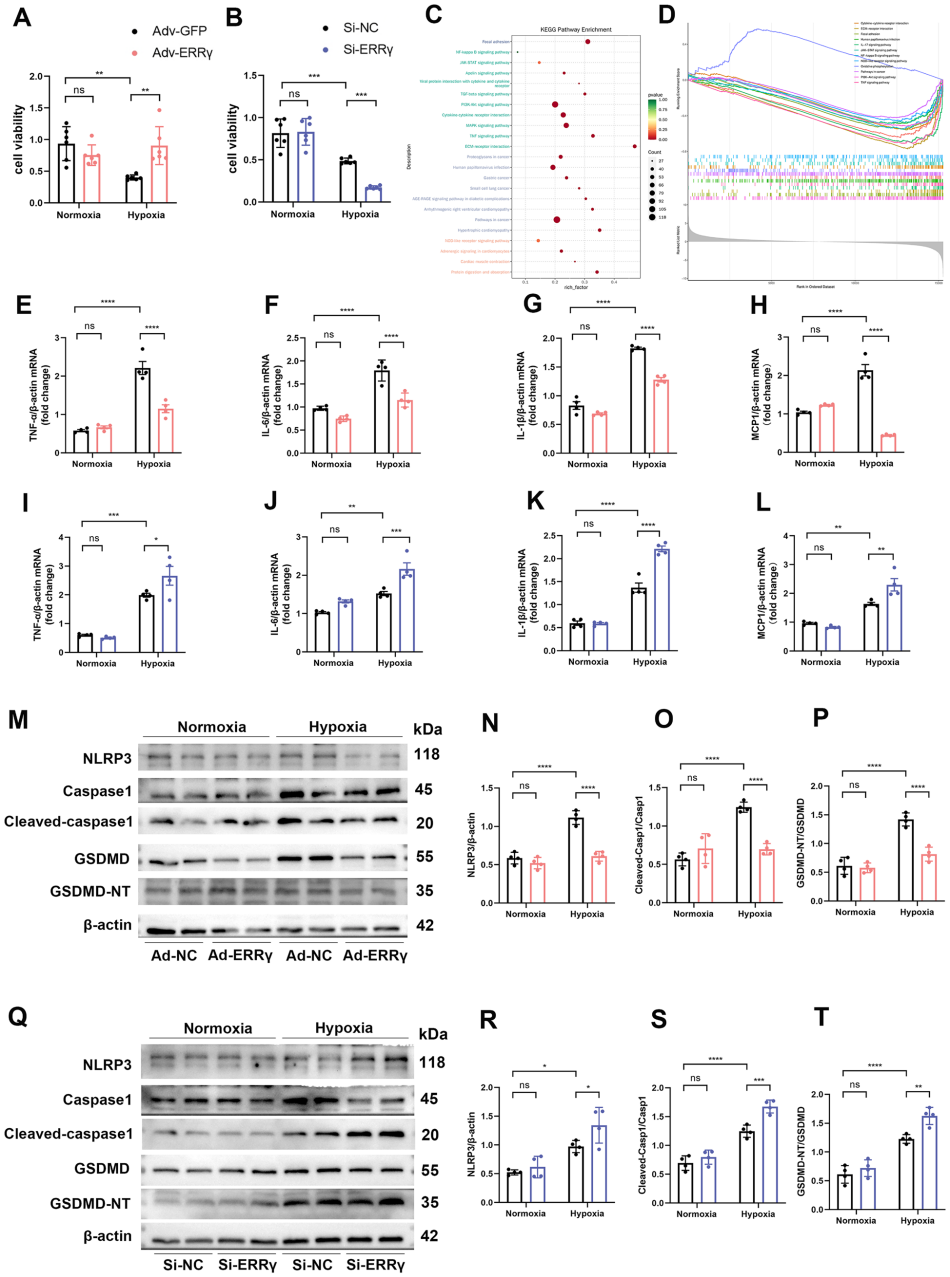
ERRγ reduces inflammation and pyroptosis in hypoxic cardiomyocytes. (A, B) CCK8 assay of cell viability in Adv‐ERRγ or Si‐ERRγ NMVMs post‐hypoxia. (C, D) KEGG and GSEA analyses of RNA‐seq data from Adv‐ERRγ and Adv‐GFP infected NMVMs under hypoxic conditions. (E–H) NMVMs were transfected with Adv‐GFP or Adv‐ERRγ for 48 h, followed by hypoxia. mRNA levels of TNF‐α, IL‐6, IL‐1β, and MCP1 were measured by qRT‐PCR. *n* = 4. (I–L) mRNA levels of TNF‐α, IL‐6, IL‐1β, and MCP1 in Si‐NC or Si‐ERRγ NMVMs under hypoxia. (M–P) Representative Western blots and quantification of NLRP3, Caspase1, cleaved‐caspase1, GSDMD, and GSDMD‐NT in NMVMs after Adv‐GFP or Adv‐ERRγ transfection and hypoxic exposure. *n* = 4. (Q–T) Western blot results of the same proteins in Si‐GFP or Si‐ERRγ NMVMs under hypoxia. Data are shown as mean ± SD. Data in (A,B, E–L, N–P, R–T) were analyzed by one‐way ANOVA with Bonferroni's multiple comparison test. *p < 0.05, **p < 0.01, ***p < 0.001, and ****p < 0.0001.

We then investigated the effects of ERRγ overexpression and knockdown in neonatal mouse ventricular myocytes (NMVMs) under hypoxic conditions. ERRγ‐adenovirus successfully increased it while ERRγ‐siRNA effectively reduced ERRγ expression in NMVMs (Figure S1A,B). NMVMs were infected with Adv‐ERRγ or Adv‐NC for 48 h and subjected to 12 h of glucose‐deprived hypoxia. Cell viability was then assessed using CCK8. Results indicated that ERRγ overexpression significantly enhanced cell viability under hypoxic conditions compared to the Adv‐NC group (Figure 3A). Conversely, knockdown of ERRγ with Si‐ERRγ resulted in significantly decreased cell viability under the same conditions (Figure 3B).

To further explore the mechanisms by which ERRγ attenuates hypoxic damage in cardiomyocytes, we performed RNA‐seq analysis on hypoxic NMVMs, and NMVMs infected with Adv‐ERRγ or Adv‐NC under hypoxic conditions. Differentially expressed genes were defined by |Log2FoldChange| > 3 and *p*‐values < 0.05. RNA‐seq data confirmed that ERRγ overexpression led to the upregulation of 188 genes and downregulation of 210 genes. Kyoto Encyclopedia of Genes and Genomes (KEGG) pathway enrichment analysis revealed enrichment in inflammation‐related pathways, including the “TNF signaling pathway,” “NF‐kappa B signaling pathway,” and “NOD‐like receptor signaling pathway” post hypoxia (Figure 3C). All enriched inflammation‐related pathways post hypoxia were downregulated by ERRγ overexpression according to Gene Set Enrichment Analysis (GSEA) (Figure 3D), suggesting that ERRγ overexpression reduces inflammation in hypoxic cardiomyocytes.

We validated several inflammatory and chemokine factors in NMVMs. RT‐qPCR indicated that, compared to Adv‐NC, ERRγ overexpression downregulated various pro‐inflammatory cytokines, including TNF‐α, IL‐6, IL‐1β, and MCP1 under hypoxia (Figure 3E–H), while ERRγ knockdown resulted in the opposite results (Figure 3I–L). Given that pyroptosis can affect inflammation levels, we also assessed the relationship between ERRγ and pyroptosis. Western blot analysis confirmed that in NMVMs overexpressing ERRγ, NLRP3 protein levels were significantly decreased, along with reduced cleavage of Caspase‐1 and GSDMD after 12 h of hypoxia (Figure 3M–P). In contrast, ERRγ knockdown NMVMs induced increased NLRP3 expression and enhanced cleavage of Caspase‐1 and GSDMD under hypoxia (Figure 3Q–T). These findings suggest that overexpression of ERRγ could mitigate inflammation and pyroptosis caused by hypoxia in cardiomyocytes.

### 3.4 ERRγ Overexpression Suppresses Inflammation and Pyroptosis After Myocardial Infarction in Mice

**FIGURE 4:**
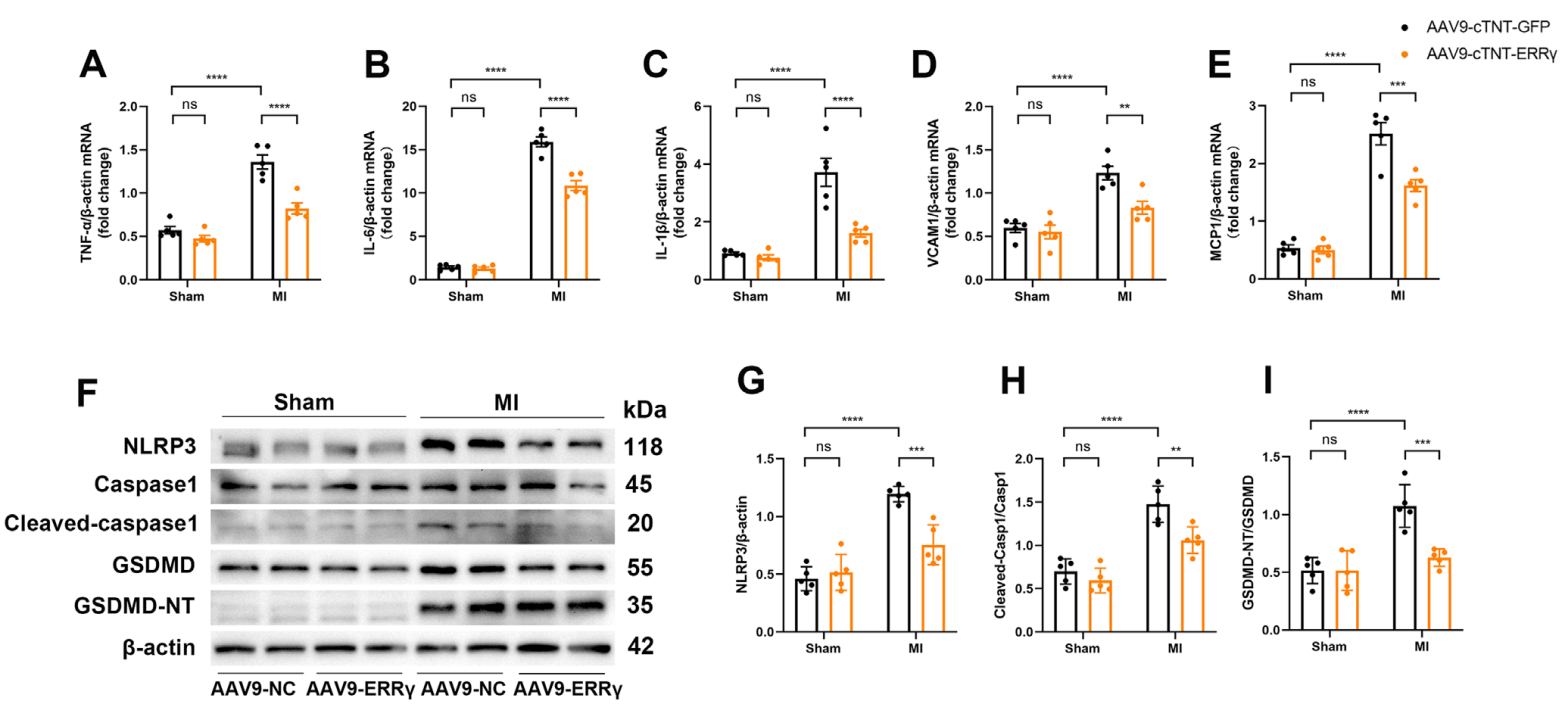
ERRγ alleviates inflammation and pyroptosis post‐MI. Mice were treated with AAV9‐cTnT‐GFP or AAV9‐cTnT‐ERRγ 4 weeks prior to sham or LAD ligation, followed by sacrifice at 3 days post‐MI. (A–E) qRT‐PCR analysis of TNF‐α, IL‐6, IL‐1β, VCAM‐1, and MCP1 mRNA levels in infarcted and healthy hearts. *n* = 5. (F–I) Representative Western blots and quantification of NLRP3, Caspase1, cleaved‐caspase1, GSDMD, and GSDMD‐NT in the border zone. *n* = 5. Data are shown as mean ± SD. Data in (A–E, G–I) were analyzed by one‐way ANOVA with Bonferroni's multiple comparison test. **p < 0.01, ***p < 0.001, and ****p < 0.0001.

We next validated the role of ERRγ in inhibiting inflammation and pyroptosis after MI in vivo. RT‐qPCR results indicated that in MI mice overexpressing ERRγ, multiple inflammatory factors, including TNF‐α, IL‐6, IL‐1β, VCAM1, and MCP1, were significantly downregulated compared to AAV9‐cTNT ‐ GFP MI mice (Figure 4A–E). Additionally, markers of pyroptosis, such as NLRP3 activation, along with the cleavage of Caspase‐1 and GSDMD, were significantly suppressed in MI mice with ERRγ overexpression (Figure 4F–I). Collectively, these findings suggest that cardiac‐specific overexpression of ERRγ alleviates inflammation and pyroptosis after MI.

### 3.5 ERRγ Overexpression Suppresses Pro‐Inflammatory Molecule GBP5 Expression in Hypoxic Cardiomyocytes

**FIGURE 5:**
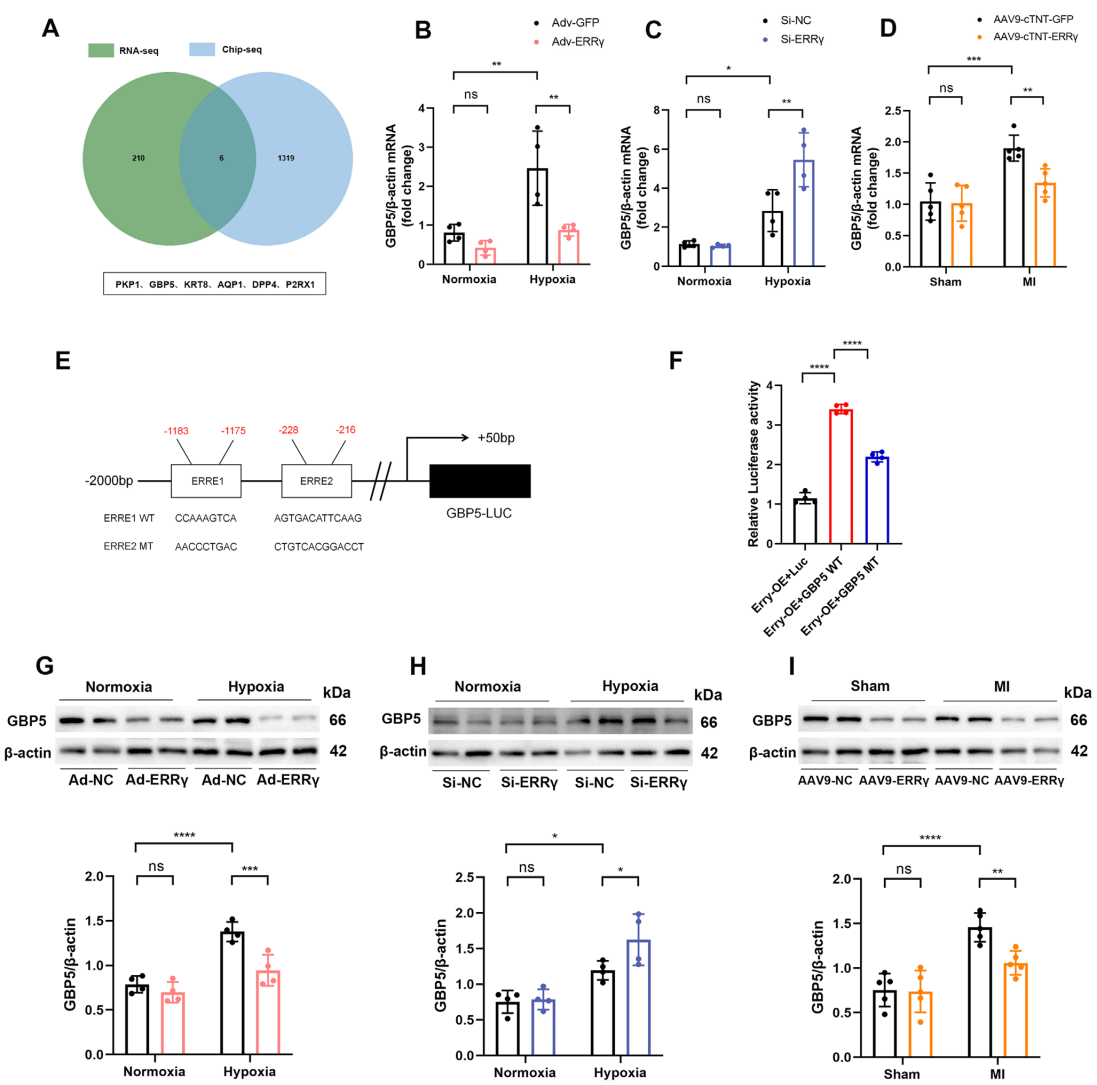
ERRγ negatively regulates GBP5 in cardiomyocytes. (A) Intersection analysis of downregulated DEGs from RNA‐seq and ChIP‐seq of ERRγ. (B–D) Effects of ERRγ overexpression or knockdown on GBP5 mRNA levels in vitro (*n* = 4) and in vivo (*n* = 5). (E) Schematic of GBP5 promoter region mutation strategy (−2000 to +50 bp). (F) Dual‐luciferase reporter assay demonstrating ERRγ binding to the GBP5 promoter to inhibit its transcription. (G–I) Effects of ERRγ overexpression or knockdown on GBP5 protein levels in vitro (*n* = 4) and in vivo (*n* = 5). Data are shown as mean ± SD. Data in (B–D, G–I, F) were analyzed by one‐way ANOVA with Bonferroni's multiple comparison test. *p < 0.05, **p < 0.01, ***p < 0.001, and ****p < 0.0001.

To further identify key downstream molecules of ERRγ mediated impact on inflammation and pyroptosis following ischemic injury, we performed an intersection analysis of downregulated differentially expressed genes (DEGs) and the ERRγ chromatin immunoprecipitation sequencing (ChIP‐seq) dataset [GSE113784]. This analysis revealed six downregulated genes (“PKP1,” “GBP5,” “KRT8,” “AQP1,” “DPP4,” and “P2RX1”) that may be directly regulated by ERRγ (Figure 5A). Among these, only GBP5 was found to be associated with inflammation and pyroptosis based on previous studies [18, 21]. Therefore, we focused on GBP5 in the subsequent investigation. RT‐qPCR analysis in NMVMs with either overexpression or knockdown of ERRγ demonstrated that under hypoxic conditions, overexpression of ERRγ led to a significant reduction in GBP5 mRNA levels (Figure 5B), whereas knockdown of ERRγ resulted in a marked increase in GBP5 mRNA levels (Figure 5C). Similarly, in vivo experiments demonstrated a reduction in GBP5 mRNA levels in MI mice overexpressing ERRγ (Figure 5D).

To confirm the direct regulatory role of ERRγ on GBP5 in cardiomyocytes, we co‐transfected GBP5 promoter constructs (GBP5‐WT‐Luc, GBP5‐MT‐Luc) with an ERRγ expression plasmid into 293 T cells. The luciferase reporter assay revealed that transfection of GBP5‐WT‐Luc significantly enhanced luciferase promoter activity, while transfection of GBP5‐MT‐Luc significantly reduced it (Figure 5E,F), strongly suggesting that ERRγ directly binds to the GBP5 promoter and induces its transcription. Subsequently, western blot analysis further confirmed that under hypoxic conditions, the expression of GBP5 was significantly downregulated in NMVMs overexpressing ERRγ (Figure 5G). Conversely, GBP5 expression was notably upregulated in ERRγ‐knockdown NMVMs (Figure 5H). The same results were observed in MI mice overexpressing ERRγ (Figure 5I). These findings demonstrate that ERRγ likely suppresses inflammation and pyroptosis by inhibiting GBP5 expression.

### 3.6 GBP5 is Essential for Mediating the Inhibitory Effects of ERRγ Overexpression on Inflammation and Pyroptosis in Hypoxic Cardiomyocytes

**FIGURE 6:**
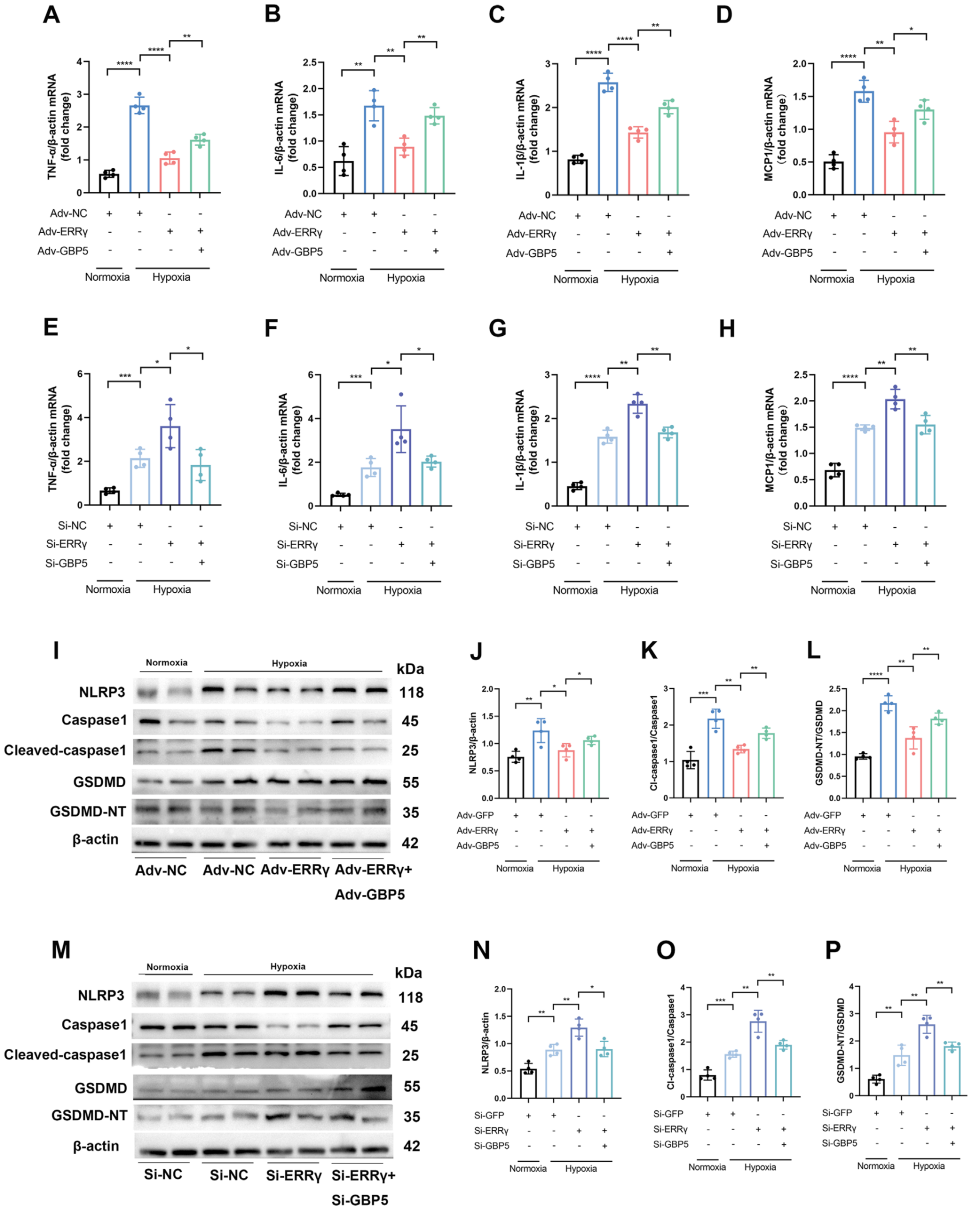
GBP5 reverses the inhibitory effects of ERRγ on inflammation and pyroptosis in cardiomyocytes. (A–D) mRNA levels of TNF‐α, IL‐6, IL‐1β, and MCP1 in ERRγ‐overexpressing NMVMs with or without GBP5 overexpression. (E–H) mRNA levels of TNF‐α, IL‐6, IL‐1β, and MCP1 in ERRγ‐knockdown NMVMs with or without GBP5 knockdown. (I–L) Protein levels of NLRP3, Caspase1, cleaved‐caspase1, GSDMD, and GSDMD‐NT in ERRγ‐overexpressing NMVMs with or without GBP5 overexpression. (M–P) Protein levels of the same markers in ERRγ‐knockdown NMVMs with or without GBP5 knockdown. *n* = 4. Data are shown as mean ± SD. Data in (A–H, J–L, N–P) were analyzed by one‐way ANOVA with Bonferroni's multiple comparison test. *p < 0.05, **p < 0.01, ***p < 0.001, and ****p < 0.0001.

To elucidate the causal relationship between the suppression/aggravation of inflammation and pyroptosis induced by ERRγ overexpression/knockdown as well as the role of GBP5 in hypoxic cardiomyocytes, we examined the effects of ERRγ overexpression in hypoxic NMVMs under conditions of GBP5 gain‐of‐function (via Adv‐GBP5) and loss‐of‐function (via Si‐GBP5). GBP5 overexpression reversed the suppression of inflammatory cytokines (Figure 6A–D) and reduction in pyroptosis (Figure 6I–L) induced by ERRγ overexpression in hypoxic NMVMs. Similarly, GBP5 knockdown attenuated the elevation of inflammatory cytokines (Figure 6E–H) and the exacerbation of pyroptosis (Figure 6M–P) caused by ERRγ knockdown in hypoxic NMVMs.

These results demonstrate that the causal relationship between ERRγ and GBP5 in regulating inflammation and pyroptosis in hypoxic cardiomyocytes.

### 3.7 GBP5 Mediates the Improvement of Cardiac Function Induced by ERRγ Overexpression Following MI In Vivo

**FIGURE 7:**
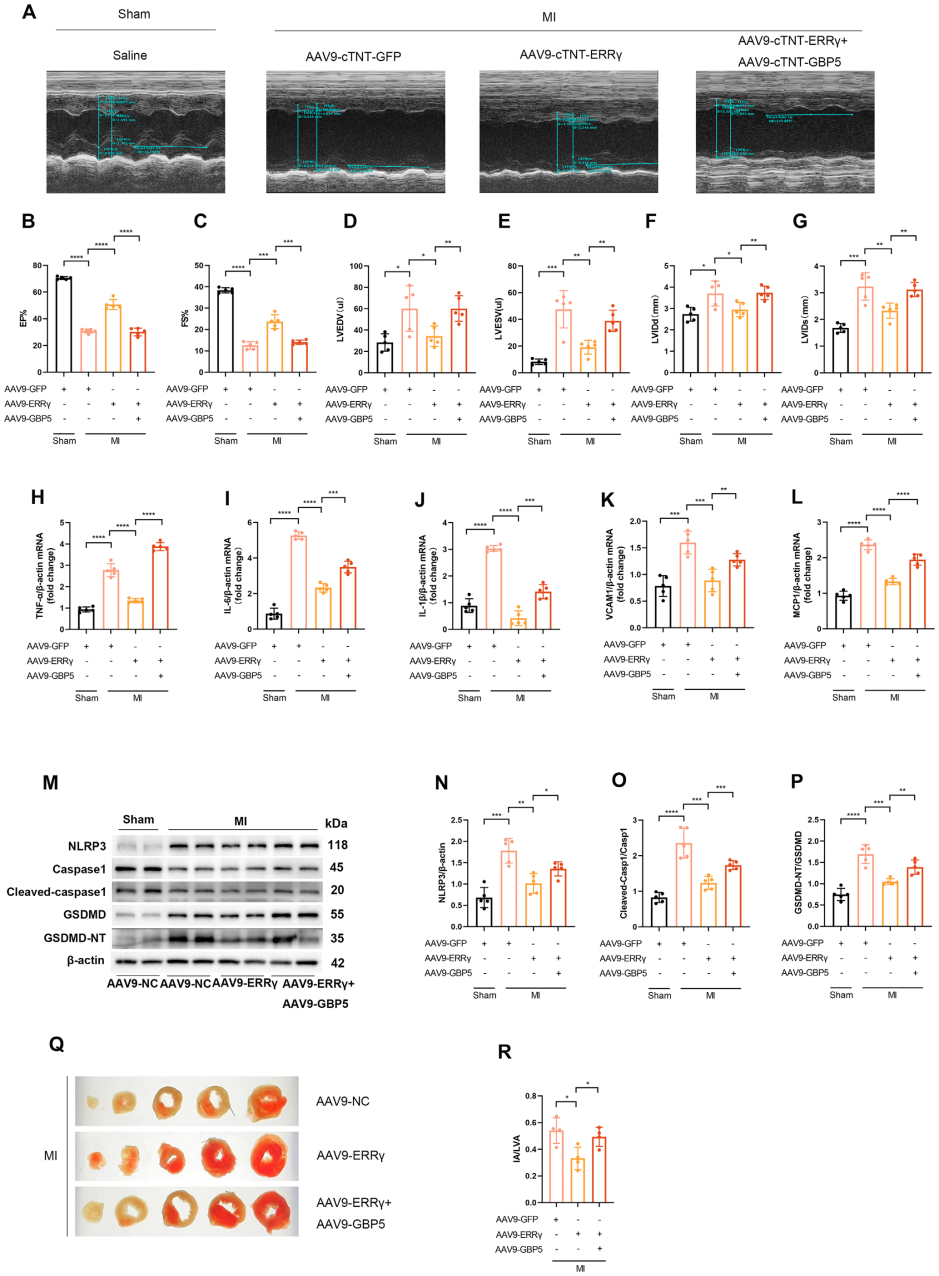
The protective role of ERRγ in post‐ischemic cardiac injury is GBP5‐dependent. (A) Representative echocardiography images of MI mice 7 days post‐infection with AAV9‐cTnT‐GFP, AAV9‐cTnT‐ERRγ, or AAV9‐cTnT‐GFP + AAV9‐cTnT‐ERRγ. (B–G) Echocardiographic analysis of cardiac function in MI mice overexpressing ERRγ with or without GBP5 overexpression. *n* = 5. (H–L) qRT‐PCR analysis of TNF‐α, IL‐6, IL‐1β, VCAM1, and MCP1 mRNA levels in ERRγ‐overexpressing mice with or without GBP5 overexpression. *n* = 5. (M–P) Western blot analysis of NLRP3, Caspase1, cleaved‐caspase1, GSDMD, and GSDMD‐NT protein levels. *n* = 5. (Q, R) Infarct size quantified by TTC staining as a percentage of ventricular area (post MI 3 day). *n* = 4. Data are shown as mean ± SD. Data in (B–L, N–P, R) were analyzed by one‐way ANOVA with Bonferroni's multiple comparison test. *p < 0.05, **p < 0.01, ***p < 0.001, and ****p < 0.0001.

We then investigated whether GBP5 mediates the cardioprotective effects of ERRγ overexpression against ischemic injury in vivo. AAV9‐ cTNT‐GBP5 was administered via tail vein injection 2 weeks after the tail vein injection of AAV9‐ cTNT‐ERRγ in mice. Four weeks later, after AAV9‐ cTNT‐GBP5 was administered, left anterior descending (LAD) coronary artery ligation was performed. The efficiency of GBP5 was shown in Figure S1D. Echocardiography examination indicated that increased GBP5 expression reversed the cardiac function improvement by overexpressing ERRγ in MI mice (Figure 7A–G).

Furthermore, inflammation was markedly exacerbated in the AAV9‐ERRγ + AAV9‐GBP5 MI mice. RT‐qPCR analysis evidenced elevated mRNA levels of TNF‐α, IL‐6, IL‐1β, VCAM1, and MCP1 in the AAV9‐ERRγ + AAV9‐GBP5 MI mice compared to AAV9‐ERRγ MI mice (Figure 7H–L). We then examined macrophage infiltration following MI. Immunofluorescence co‐staining of cTNT and F4/80 in the infarct border zone revealed that cardiomyocyte‐specific overexpression of ERRγ reduced the accumulation of F4/80^+^ macrophages in the infarct border zone on Day 3 post‐MI. However, this inhibitory effect was reversed when ERRγ was co‐overexpressed with GBP5 (Figure S1E). Similarly, pyroptosis changes followed the same trend (Figure 7M–P). During pyroptosis, the release of inflammatory cytokines depends on the formation of pyroptotic pores on the cardiomyocyte membrane [22]. Transmission electron microscopy showed that overexpression of ERRγ reduced ischemia‐induced membrane pore formation, whereas co‐overexpression of ERRγ and GBP5 blocked these beneficial effects induced by ERRγ overexpression (Figure S1F). TTC staining results showed that the reduction in infarct size post ERRγ overexpression was reversed in AAV9‐ERRγ + AAV9‐GBP5 MI mice (Figure 7Q,R).

These results demonstrate that the inhibition of MI‐induced inflammation and pyroptosis by ERRγ, leading to improved cardiac function, is mediated through the downregulation of GBP5 expression.

### 3.8 ERRγ Selective Agonist, DY131, Suppressed Inflammation and Reduced Cardiac Ischemic Injury Post‐MI

**FIGURE 8:**
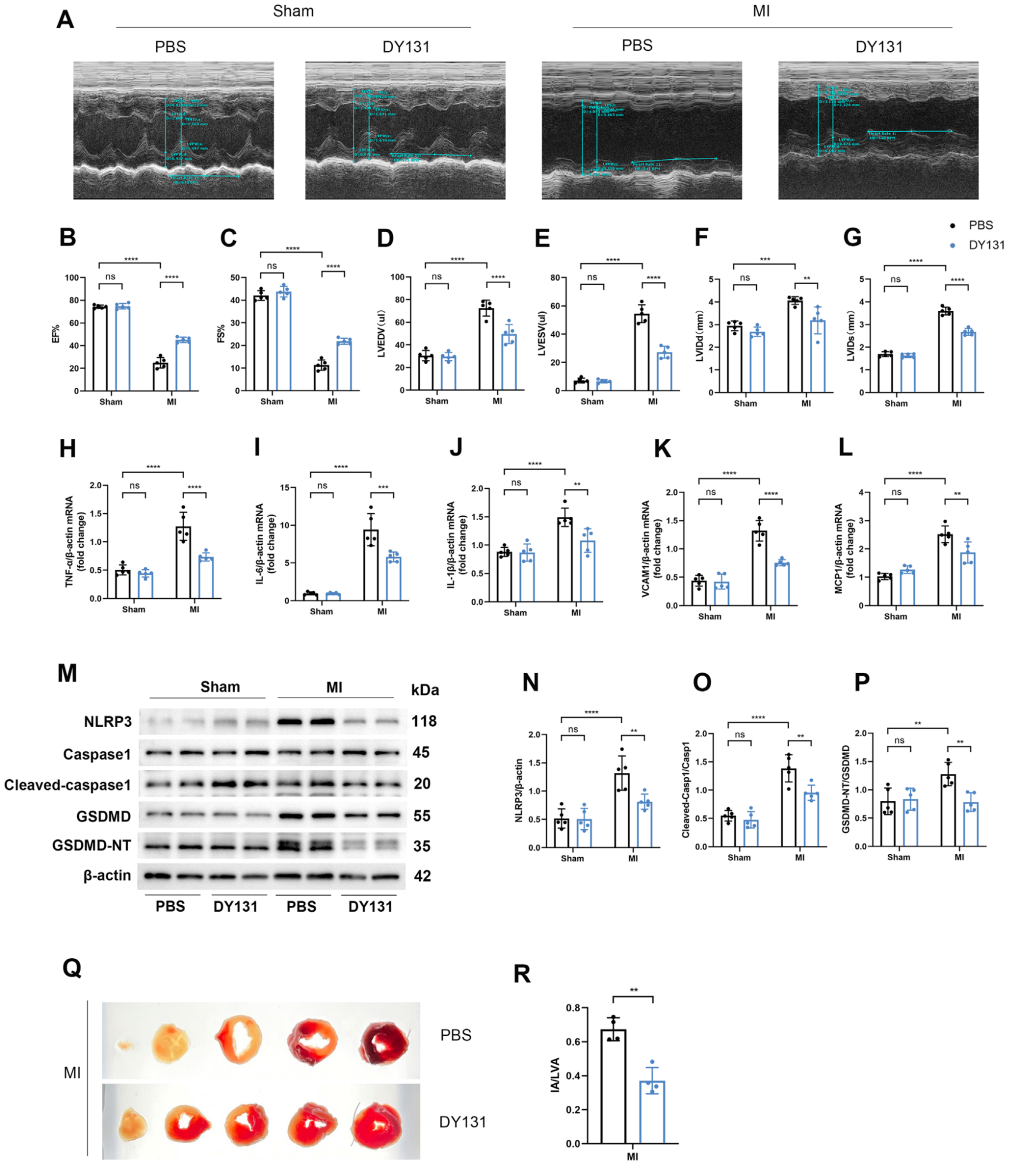
DY131 mimics ERRγ‐mediated anti‐inflammatory effects post‐MI. (A) Representative echocardiography images of MI mice treated with DY131 (10 mg/kg) or PBS by intraperitoneal injection at 7 days post‐MI. (B–G) Echocardiographic analysis of cardiac function in DY131 or PBS‐treated MI mice. *n* = 5. (H–L) qRT‐PCR analysis of TNF‐α, IL‐6, IL‐1β, VCAM1, and MCP1 mRNA levels in DY131‐ or PBS‐treated MI mice. *n* = 5. (M–P) Western blot analysis of NLRP3, Caspase1, cleaved‐caspase1, GSDMD, and GSDMD‐NT protein levels. *n* = 5. (Q, R) Infarct size quantified by TTC staining as a percentage of ventricular area (post MI 3 day). *n* = 4. Data are shown as mean ± SD. Data in (B–L, N–P) were analyzed by one‐way ANOVA with Bonferroni's multiple comparison test. Data in R were analyzed by two‐tailed Student's *t*‐test. **p < 0.01, ***p < 0.001, and ****p < 0.0001.

DY131, a specific agonist of ERRγ, has been shown to play a critical role in the regulation of apoptosis, inflammation, oxidative stress, and energy metabolism [14]. Herein, we aimed to investigate whether DY131 could also alleviate ischemic injury following MI. Mice were pre‐treated with DY131 before being subjected to LAD ligation. Echocardiographic assessment indicated that MI mice pre‐treated with DY131 showed significant improvements in cardiac function compared to PBS‐pretreated MI mice (Figure 8A–G).

As expected, mRNA levels of TNF‐α, IL‐6, IL‐1β, VCAM1, and MCP1, which were elevated in PBS‐pretreated MI mice, were all significantly reduced in DY131‐pretreated MI mice (Figure 8H–L). In addition, NLRP3 activation, as well as the cleavage of Caspase‐1 and GSDMD, was markedly suppressed in DY131‐pretreated MI mice (Figure 8M–P). TTC staining also revealed that DY131 pre‐treatment significantly reduced infarct size in MI mice (Figure 8Q,R).

In summary, these results suggest that DY131 has the potential to ameliorate MI‐induced cardiac dysfunction by mitigating inflammation and pyroptosis.

## 4 Discussion

In this study, we identified the significant protective role of ERRγ overexpression and selective ERRγ agonist DY131 in reducing myocardial injury following MI by mitigating inflammation and pyroptosis through downregulating GBP5. In detail, ERRγ binds to the GBP5 promoter, suppressing its expression and inhibiting NLRP3 inflammasome assembly, thereby reducing myocardial inflammation and pyroptosis post‐MI. Our study thus highlights the critical role of ERRγ in regulating the inflammatory response in cardiomyocytes post‐MI, and ERRγ activation might serve as a promising therapeutic option for MI treatment. To our best knowledge, this is the first mechanistic study revealing the role of ERRγ in the setting of ischemic injury.

ERRγ is a member of the nuclear receptor family, known as an orphan nuclear receptor due to the absence of an endogenous ligand [11]. Once activated, ERRγ regulates various cellular processes through gene expression modulation [23]. While ERRγ plays a significant role in cardiovascular diseases, its specific function in the heart remains unclear. Previous studies have shown that ERRγ expression increases in patients with hypertrophic cardiomyopathy and in animal models of cardiac hypertrophy, where cardiomyocyte‐specific overexpression of ERRγ induces hypertrophic phenotypes [24]. In diabetic mice, elevated ERRγ expression promotes the expression of genes related to lipid and palmitate oxidation, leading to metabolic disturbances in cardiomyocytes and contributing to diabetic cardiomyopathy [25]. Moreover, Sakamoto et al. demonstrated that ERRγ suppresses embryonic lineage gene expression while promoting the expression of genes involved in postnatal maturation, including mitochondrial energy transduction, contractile function, and ion transport [26]. However, these studies focus on non‐ischemic cardiovascular diseases. Thus, the role of ERRγ in ischemic cardiomyopathy remains undefined. Our research uncovers a previously unrecognized role of ERRγ in ischemic heart disease. Unlike the response to pressure overload and metabolic stress, ERRγ expression is significantly downregulated in cardiomyocytes subjected to ischemic or hypoxic stress, with the most pronounced decrease occurring during the early phase of MI. Additionally, our data reveal that cardiomyocyte‐specific overexpression of ERRγ significantly attenuates early myocardial inflammation and pyroptosis, improving adverse cardiac remodeling post‐MI. Furthermore, we identified GBP5 as a target through which ERRγ exerts its anti‐inflammatory effects.

Post‐MI cardiac repair involves a series of tightly regulated events, primarily encompassing an inflammatory phase followed by a proliferative repair phase. Early activation of inflammation is crucial for transitioning to the subsequent repair phase; however, excessive or prolonged inflammation can lead to myocardial tissue damage, impaired scar formation, and ultimately, compromised cardiac contractile function [27]. Substantial progress has been made in understanding the regulation of inflammatory responses in cardiomyocytes following MI. Under persistent hypoxic stress, necrotic cardiomyocytes release DAMPs, which act as key stimuli, triggering inflammatory pathways and promoting cytokine release [28, 29]. The surviving cardiomyocytes in the infarct border zone become focal points for immune cell recruitment, where neutrophils and monocytes migrate and activate under the influence of chemokines and cytokines to clear necrotic cells and matrix debris in the infarcted heart [30]. Furthermore, GSDMD‐mediated pyroptosis in cardiomyocytes is considered a pivotal event in myocardial ischemia [7]. Pyroptosis, an inflammation‐associated form of programmed cell death, can be initiated by various caspases and inflammasome pathways. Previous studies have shown that RNA‐seq analysis of the left ventricle within 0–72 h post‐acute MI reveals significant enrichment of pyroptosis pathways in 24‐h and 72‐h AMI samples compared to sham samples, alongside elevated levels of Caspase1 and GSDMD protein as AMI progresses [31, 32]. Moreover, pre‐treatment of AMI mice with the pyroptosis inhibitor VX‐765 significantly improved cardiac function and reduced infarct size [31]. Our study extends these findings by demonstrating that: [1] Transcriptomic analysis revealed that ERRγ negatively regulates inflammation‐related pathways, including the TNF‐α signaling pathway and NOD‐like receptor signaling pathway. We confirmed that cardiomyocyte‐specific overexpression of ERRγ in vivo and in vitro significantly suppresses the mRNA levels of pro‐inflammatory cytokines (TNF‐α, IL‐6, IL‐1β, MCP1, VCAM1) [2]. Immunoblotting assays further demonstrated that ERRγ inhibits pyroptosis under ischemic and hypoxic conditions, thereby improving cardiac function and reducing infarct size post‐MI. This is the first study to identify cardiomyocyte ERRγ as a regulator of cardiomyocyte fate and a suppressor of myocardial inflammation.

In addition to the orphan nuclear receptor ERRγ, other nuclear receptors also play significant roles in MI. The peroxisome proliferator‐activated receptors (PPARs), belonging to the nuclear hormone receptor family, are ligand‐activated transcription factors with three distinct isoforms: PPARα (NR1C1), PPARβ/δ (NR1C2), and PPARγ (NR1C3), all of which exert varying degrees of influence during myocardial ischemia [33]. Tian‐Li Yue and colleagues demonstrated that the PPARα agonist GW7647 attenuated the reduction in myocardial fatty acid oxidase activity, mitigated pro‐inflammatory cytokine release, reduced neutrophil infiltration, and suppressed NF‐κB signaling activation in a murine ischemia–reperfusion model, thereby improving cardiac contractility and reducing infarct size [34]. Similarly, studies have shown that although the PPARα agonist clofibrate did not significantly enhance left ventricular ejection fraction in a rat MI model, it effectively suppressed the release of pro‐inflammatory cytokines (e.g., IL‐6, TNFα, ICAM1, and VCAM1) [33]. Magadum et al. reported that cardiomyocyte‐specific overexpression of PPARβ/δ led to reduced infarct size, enhanced cardiomyocyte proliferation, and improved cardiac function. Furthermore, they observed that treatment with the PPARβ/δ agonist GW0742 after left anterior descending coronary artery ligation in mice produced comparable cardioprotective effects [35]. Other studies have indicated that the PPARγ agonist rosiglitazone reduced infarct size and improved cardiac function in ischemia–reperfusion rats by diminishing macrophage and neutrophil infiltration as well as suppressing inflammatory cytokine release [36].

Moreover, extensive evidence suggests that estrogen receptors (ERs) confer cardioprotection via anti‐apoptotic, anti‐inflammatory, and antioxidant mechanisms [37]. In female mice, ERα overexpression improved post‐MI cardiac function, downregulated the expression of type I and III collagen genes, reduced collagen deposition, and induced phosphorylation of the JNK signaling pathway. Additionally, ERα overexpression enhanced angiogenesis, lymphangiogenesis, and neovascularization in the peri‐infarct zone of both female and male mice [38]. Treatment with the ERα agonist propyl‐pyrazole‐triol (PPT) exerted cardioprotective effects in MI mice [37]. Studies also revealed that ERβ‐overexpressing mice exhibited reduced post‐MI collagen deposition, increased LVEF and fractional shortening, and improved cardiac function compared to wild‐type controls [39]. Pre‐treatment with the ERβ agonist diarylpropionitrile (DPN) prior to MI induction in mice reduced infarct size and serum cardiac enzyme levels while protecting cardiomyocytes against oxidative stress and apoptosis [40].

In our experiments, we found that ERRγ suppressed GBP5 expression, thereby inhibiting NLRP3 inflammasome assembly, attenuating cardiac inflammation and pyroptosis, and ultimately improving cardiac function—suggesting that ERRγ, like PPARs, mitigates post‐MI inflammation to enhance cardiac performance. Furthermore, pre‐treatment with the ERRγ agonist DY131 in MI mice alleviated post‐MI inflammatory responses and pyroptosis while ameliorating left ventricular systolic dysfunction.

Increasing evidence indicates that GBP5, a key member of the GTPase family, plays a critical role in inflammation and pyroptosis across various tissues. In osteoarthritis, GBP5 expression is significantly upregulated in TNF‐α‐treated chondrocytes, where IRF1 promotes GBP5 transcription, triggering NLRP3 inflammasome activation and promoting pyroptosis [21]. In sepsis‐associated liver injury, GBP5 overexpression aggravates liver damage by promoting the release of inflammatory cytokines through NLRP3 inflammasome activation [18]. Additionally, BMP7 alleviates pyroptosis and inflammation and improves cardiac dysfunction in diabetic cardiomyopathy by inhibiting the Nek7/GBP5 signaling axis, thus suppressing inflammasome formation in cardiomyocytes [41]. In our study, we identified GBP5 as a gene related to inflammation and pyroptosis through intersecting ERRγ RNA‐seq and ChIP‐seq data (GSE113760) with literature review. We validated the negative correlation between ERRγ and GBP5 at both the mRNA and protein levels in cardiomyocytes using qPCR and Western blot. Notably, dual luciferase assays demonstrated that ERRγ binds to the GBP5 promoter region, suggesting that ERRγ negatively regulates GBP5 expression. Moreover, GBP5 overexpression reversed the inhibitory effects of ERRγ on inflammation and pyroptosis in hypoxic cardiomyocytes, establishing for the first time the regulatory relationship between ERRγ and GBP5 in cardiomyocytes.

In our supplementary experiments, we further evaluated macrophage infiltration and cardiomyocyte pyroptosis in the infarct border zone at Day 3 post‐MI. F4/80, a well‐established surface marker for macrophages [28], was used for immunofluorescence staining. Since Day 3 post‐infarction represents the peak of the inflammatory response, we observed that the F4/80 fluorescence intensity was markedly reduced in the infarct border zone of AAV9‐ERRγ MI mice compared to AAV9‐GFP MI controls. However, this reduction was reversed in AAV9‐ERRγ + AAV9‐GBP5 MI mice, in which F4/80 signal intensity was increased. These findings suggest that cardiomyocyte‐specific overexpression of ERRγ attenuates macrophage infiltration and thus suppresses the inflammatory response, whereas GBP5 reconstitution abolishes the anti‐inflammatory effect of ERRγ. This provides further support that GBP5 functions downstream of ERRγ in mediating its anti‐inflammatory effects. Similarly, pyroptosis plays a critical role in myocardial injury at this early stage [31]. Transmission electron microscopy (TEM), a powerful tool for assessing cellular morphology and ultrastructure, revealed a reduction in pyroptotic features—such as plasma membrane permeabilization, organelle swelling, and cytoplasmic granulation or vacuolization—in the infarct border zone of AAV9‐ERRγ MI mice compared to AAV9‐GFP controls. In contrast, pyroptotic characteristics were exacerbated in AAV9‐ERRγ + AAV9‐GBP5 MI mice. These TEM observations further support the notion that ERRγ overexpression in cardiomyocytes suppresses MI‐induced pyroptosis, and that GBP5 mediates this protective effect as a downstream effector. Collectively, these results reinforce the dual role of ERRγ in mitigating post‐MI myocardial inflammation and pyroptosis, and highlight GBP5 as a key downstream mediator of ERRγ function.

DY131, an ERRγ agonist, has shown protective effects in various disease models. It mitigates mitochondrial dysfunction and metabolic disturbances in renal tubular epithelial cells, reducing tubular injury in acute kidney injury [13]. In LPS‐induced acute liver injury, DY131 decreases oxidative stress and inflammation, thus alleviating liver damage [14]. In our study, MI mice pretreated with DY131 showed significantly improved cardiac function and reduced infarct size compared to PBS‐pretreated MI mice. Additionally, DY131 pretreatment led to decreased levels of inflammatory cytokines and pyroptosis. These findings suggest that targeting the ERRγ/GBP5 axis may be a promising therapeutic strategy for MI, and DY131 could serve as a potential novel therapeutic agent for ischemic cardiomyopathy. To date, no ERRγ agonists have been approved for clinical use, nor have clinical trials demonstrated their efficacy in MI; current research remains confined to animal studies. In these preclinical investigations, DY131 was predominantly administered intraperitoneally to examine its effects in LPS‐induced acute liver injury [14], cisplatin‐induced acute kidney injury [13], and early brain injury after subarachnoid hemorrhage [42]. Subcutaneous injection was employed to evaluate DY131's role in gastric cancer xenograft models [43]. Previous studies indicated that low‐dose intraperitoneal DY131 administration caused no significant hepatic or renal toxicity in mice, with H&E and PAS staining of liver tissues showing no morphological or glycogen‐level alterations compared to controls [13, 14]. No overt toxicity or off‐target organ damage has been reported with DY131 thus far. Based on the aforementioned studies, it is reasonable to hypothesize that DY131 may be suitable for systemic administration. However, current research on DY131 remains limited to small animal models, and further investigation in large animal models is essential to comprehensively evaluate its toxicity profile, systemic suitability, and potential off‐target effects.

## Author Contributions

Junhao Qiu: conceptualized and designed the experiments. Qianji Che and Yichao Zhang: performed the experiments. Junhao Qiu: prepared the manuscript and the figures. Qianji Che, Yichao Zhang, Mu Chen, and Zhixing Wei: contributed to establishing the mice model of MI. Yangjinming Bai and Tingting Zhao: contributed to the echocardiography, TTC, and immunofluorescence experiments. Ji Yan and Zhengyang Wu: contributed to the western blot and RT‐qPCR experiments. Zhentao Fei: contributed to the statistical analyses. Yigang Li, Qian Wang, and Yuepeng Wang: reviewed and made important suggestions for the manuscript. All authors commented on previous versions of the manuscript. All authors read and approved the final manuscript.

## Conflicts of Interest

The authors declare no conflicts of interest.

## Supporting information


Data S1:

## Data Availability

The data that support the findings of this study are available in the Materials and Methods, Results, and/or Supporting Information S1 of this article.
