# Supplementary material for: Nuclear Receptor ERRγ Protects Against Cardiac Ischemic Injury by Suppressing GBP5‐Mediated Myocardial Inflammation
Source: FASEB J. 2025 Jul 10;39(14):e70819. doi: 10.1096/fj.202500763R (PMC12242898; doi:10.1096/fj.202500763R)
Supplement: Supplementary file 1 — Data S1: [file FSB2-39-e70819-s001.zip › 202500763R-sup-0003-SI_Text-S01.docx]

**Nuclear Receptor ERRγ protects against cardiac ischemic injury by suppressing GBP5-mediated myocardial inflammation**

Junhao Qiu, MS^*,1^, Qianji Che, MD^*,1^, Yichao Zhang, MD^*,1^, Mu Chen, MD^1^, Zhixing Wei, MD^1^, Yangjinming Bai, MD^1^, Tingting Zhao, MD^1^, Ji Yan, MD^1^, Zhengyang Wu, MS^1^, Zhentao Fei, MS^1^, Yuepeng Wang, MD^1^, Qian Wang, MD^1^, Yi-Gang Li ,MD^1^

^1^Department of Cardiology, Xinhua Hospital, School of Medicine, Shanghai Jiao Tong University, 1665 Kongjiang Road, Shanghai 200092, China

^*^Junhao Qiu, Qianji Che and Yichao Zhang contributed equally.

Running title: ERRγ PROTECTS CARDIAC ISCHMEIC INJURY

Corresponding author:

Yigang Li, MD, FACC FHRS

Department of Cardiology, Xinhua Hospital, School of Medicine, Shanghai Jiao Tong University, 1665 Kongjiang Rd, Shanghai 200092, China,

Phone: +86 137 6131 8166

Email: liyigang@xinhuamed.com.cn;

**Supplemental Information**

**Legend**

**Supplementary 1** ERRγ regulates macrophage activity and pyroptosome formation. (A) Verification of Si-ERRγ knockdown efficiency in NMVMs. (B) Validation of Adv-ERRγ overexpression efficiency in NMVMs. (C-D) Verification of AAV9-ERRγ and AAV9-GBP5 overexpression efficiency in mice. (E) Immunofluorescence co-staining of cTnT, F4/80, and DAPI in the infarcted hearts of mice treated with AAV9-cTnT-GFP, AAV9-cTnT-ERRγ, or AAV9-cTnT-ERRγ + AAV9-cTnT-GBP5. Scale bar = 100 μm. n=4. (F) Electron microscopy illustrating pyroptosis in AMI samples from different treatment groups. Scale bar = 2 μm. Data are shown as means ± SD. Data in E were analyzed by one-way ANOVA with Bonferroni’s multiple comparison test. * p < 0.05, ** p < 0.01, *** p < 0.001,**** p < 0.0001.
