# Supplementary figures and images for: Nuclear Receptor ERRγ Protects Against Cardiac Ischemic Injury by Suppressing GBP5‐Mediated Myocardial Inflammation
Source: FASEB J. 2025 Jul 10;39(14):e70819. doi: 10.1096/fj.202500763R (PMC12242898; doi:10.1096/fj.202500763R)

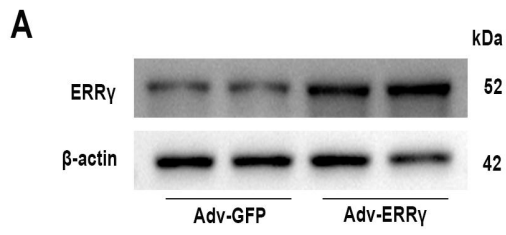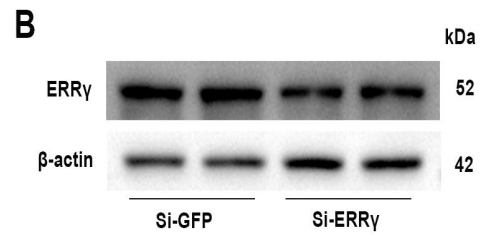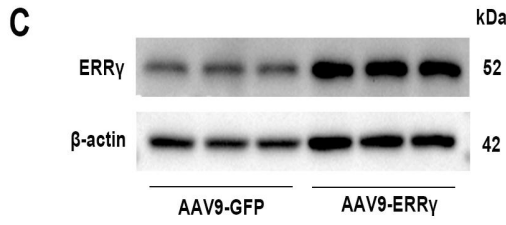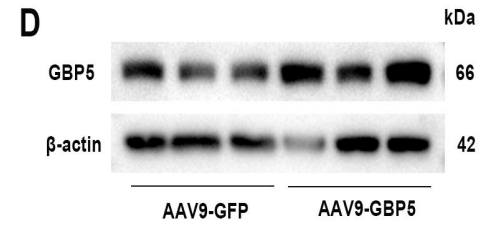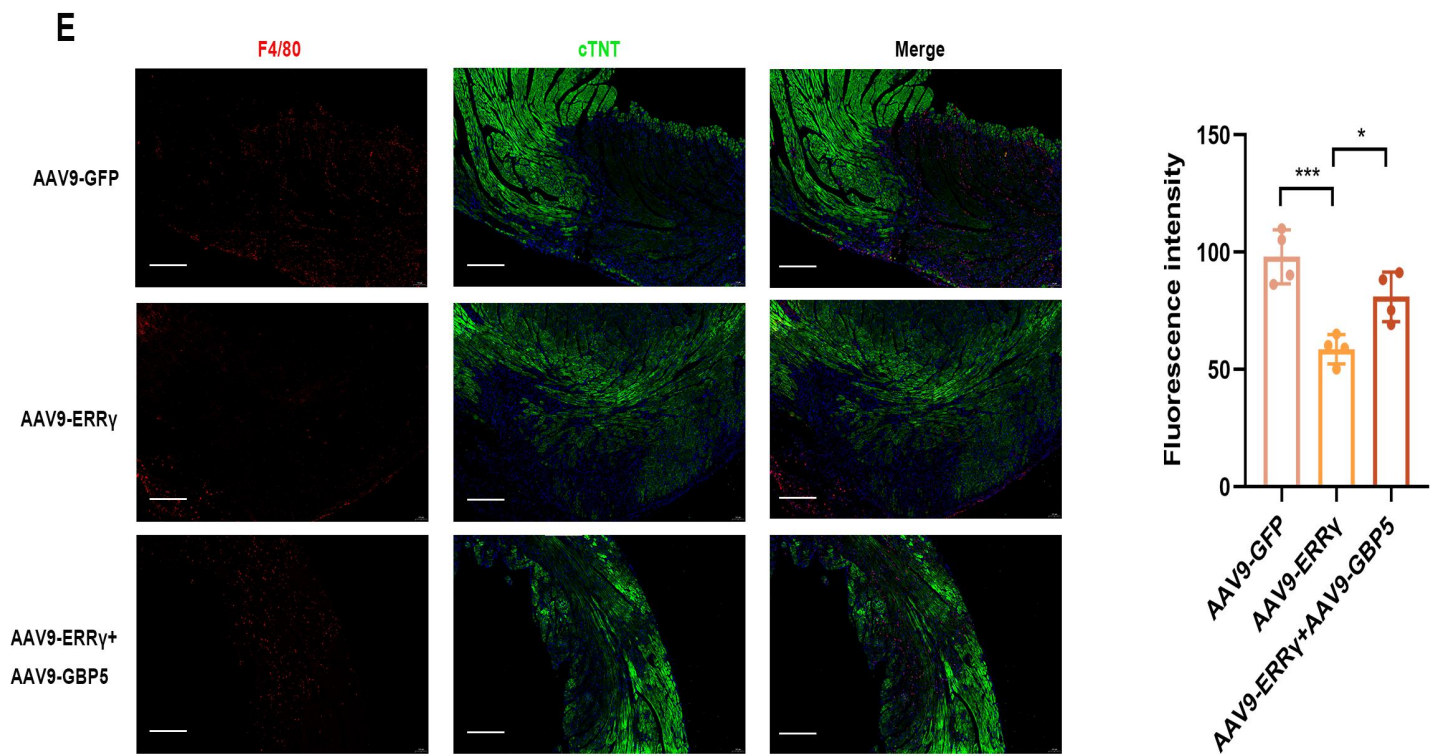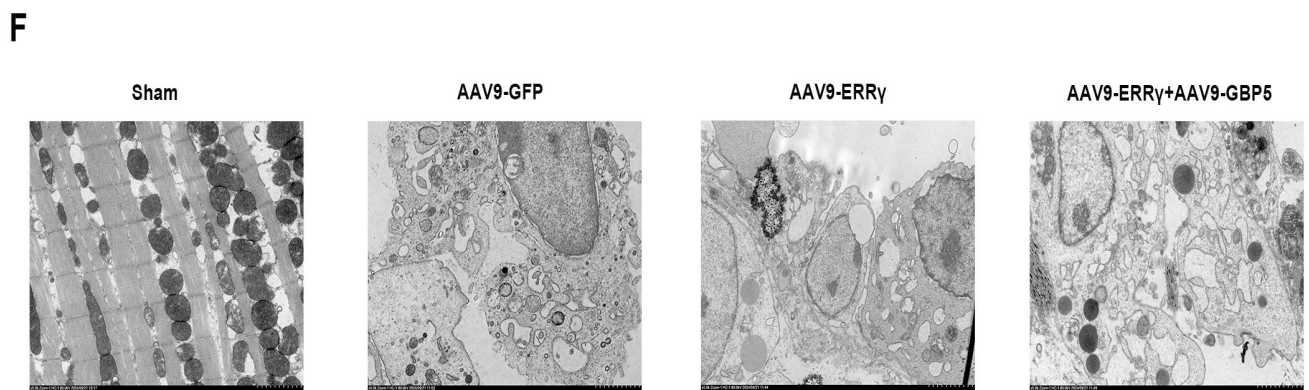

Supplement: Supplementary file 1 — Data S1: [file FSB2-39-e70819-s001.zip › 202500763R-f09-z-.pdf]
